# Heart Rate Variability and Salivary Biomarkers Differences between Fibromyalgia and Healthy Participants after an Exercise Fatigue Protocol: An Experimental Study

**DOI:** 10.3390/diagnostics12092220

**Published:** 2022-09-14

**Authors:** Ana Rodrigues Costa, Ana Freire, Jose A. Parraca, Vanda Silva, Pablo Tomas-Carus, Santos Villafaina

**Affiliations:** 1 Departamento de Ciências Médicas e da Saúde, Escola de Saúde e Desenvolvimento Humano, Universidade de Évora, 7004-516 Évora, Portugal; 2 Departamento de Desporto e Saúde, Escola de Saúde e Desenvolvimento Humano, Universidade de Évora, 7004-516 Évora, Portugal; 3 Comprehensive Health Research Centre (CHRC), University of Évora, 7004-516 Évora, Portugal; 4 Family Health Unit—Lusitania, Rua do Ferragial do Poço Novo, S/N, 7000-727 Évora, Portugal; 5 Universidad de Extremadura, Facultad de Ciencias del Deporte, 10003 Cáceres, Spain

**Keywords:** autonomic modulation, physical exercise, fatigue, sympathetic nervous system

## Abstract

Previous studies showed that people with Fibromyalgia (FM) suffer from dysautonomia. Dysautonomia consists of persistent autonomic nervous system hyperactivity at rest and hyporeactivity during stressful situations. There is evidence that parameters reflecting the complex interplay between the autonomic nervous system and the cardiovascular system during exercise can provide significant prognostic information. Therefore, this study aimed to investigate the differences between people with FM and healthy controls on heart rate variability (HRV) and salivary parameters (such as flow, protein concentration, enzymatic activities of amylase, catalase and glutathione peroxidase) in two moments: (1) at baseline, and (2) after an exercise fatigue protocol. A total of 37 participants, twenty-one were people with fibromyalgia and sixteen were healthy controls, participated in this cross-sectional study. HRV and salivary samples were collected before and after an exercise fatigue protocol. The fatigue protocol consisted of 20 repetitions of knee extensions and flexions of the dominant leg at 180 °·s^−1^ (degrees per second). Significant differences were found in the HRV (stress index, LF and HF variables) and salivary biomarkers (with a higher concentration of salivary amylase in people with FM compared to healthy controls). Exercise acute effects on HRV showed that people with FM did not significantly react to exercise. However, significant differences between baseline and post-exercise on HRV significantly induce alteration on the HRV of healthy controls. Catalase significantly increased after exercise in healthy controls whereas salivary flow significantly increased in women with FM after an exercise fatigue protocol. Our study suggests that a higher α-amylase activity and an impaired HRV can be used as possible biomarkers of fibromyalgia, associated with a reduction in salivary flow without changes in HRV and catalase activity after a fatigue exercise protocol. More studies should be carried out in the future to evaluate this hypothesis, in order to find diagnostic biomarkers in fibromyalgia.

## 1. Introduction

Fibromyalgia (FM) is characterized by chronic, widespread and persistent pain [1]. It is accompanied by several symptoms such as stiffness, sleep disorders, depression, anxiety or mobility and cognitive impairments [1,2]. It affects 2.7% of the world population, with a higher incidence in women (4.2%) than in men (1.4%) with a female/male ratio of 3:1 [1]. Although of the unknown etiology, fibromyalgia onset is sometimes associated with exposure to situations of stress or trauma, which can cause changes in the functioning of the autonomic nervous system, a feature of the disease [3,4,5,6]. Diagnostic biochemical markers for this pathology have not yet been defined [7].

The autonomic nervous system (ANS) is a component of the peripheral nervous system that involuntary regulates the function of different organs and glands (through antagonistic sympathetic and parasympathetic modulation) to maintain homeostasis. Previous studies showed that people with FM suffer from an autonomic nervous system dysfunction (dysautonomia) [8,9,10,11]. The dysautonomia consisted of persistent autonomic nervous system hyperactivity at rest and hyporeactivity during stressful situations [12]. Thus, multisystem features of FM could be explained by dysautonomia [13]. In this regard, the heart rate variability (HRV) provides information regarding the balance between sympathetic and parasympathetic modulation [14]. The HRV is a non-invasive measure of the beat-to-beat variation over an interval of time [15]. A low HRV was related to risk of death from several causes [16]. Regarding people with FM, previous studies have reported abnormal HRV that could be compatible with dysautonomia [8,9,10,11].

The imbalance between the production of reactive oxygen species (ROS) and the antioxidant capacity resulting from stress may be at the origin of the changes observed in the functioning of the ANS and of nociception in people with FM [3,17]. In people with fibromyalgia, alterations in the expression of catalase and glutathione peroxidase, both enzymes capable of converting H_2_O_2_ into H_2_O, have already been detected [18]. Saliva is a biological fluid appropriate to the study of biochemical markers for several pathologies and shows high potential in those with associated nervous system dysfunctions [7], since saliva composition depends on the stimuli acting on the main glands that produce it [19]. Salivary glands form a highly sophisticated end point in the central nervous system control of local immune defenses, capable of responding instantly and with a high level of specificity to potential sources of harm (i.e., food, stress or inflammation) [19]. The sympathetic control of salivary production is via the superior cervical ganglion. Sympathetic stimulation results in the release of noradrenaline, which acts upon α- and β-adrenergic receptors. This results in: (a) decreased production of saliva by acinar cells; (b) increased protein secretion; and (c) decreased blood flow to the glands. There is a variable sympathetic innervation between the salivary glands [20]. The parasympathetic outflow is coordinated via centers in the medulla, and innervation occurs via the facial and glossopharyngeal nerves. Afferent information from the mouth, tongue, nose, and conditioned reflexes are integrated within the brain—and in the presence of food, parasympathetic stimulation occurs [21]. Parasympathetic outflow results in the release of acetylcholine (ACh) onto M3 muscarinic receptors. This results in: (a) acinar cells increase saliva secretion; (b) duct cells increase HCO3 secretion; (c) co-transmitters result in increased blood flow to the salivary glands; (d) contraction of myoepithelium to increase the rate of saliva expulsion [21].

There is evidence that parameters reflecting the complex interplay between the autonomic nervous system and the cardiovascular system during exercise can provide significant prognostic information [14]. Thus, exercise has been used to study the ANS response of people with FM. Schamne, et al. [22] performed a maximal incremental exercise in women with FM measuring the cardiac autonomic response through the HRV. Results showed an abnormal autonomic modulation at rest, during, and after exercise in people with FM. Similarly, Kingsley, et al. [23] showed that people with FM exhibited an altered autonomic modulation pattern after resistance exercise compared to healthy controls. However, the acute effects of exercise on salivary biochemical parameters have been poorly studied in people with FM. Furthermore, physical exercise is a strong activator of the sympathetic nervous system, altering the REDOX status in healthy individuals and patients [3]. The application of a single moment of intense exercise in sedentary individuals with fibromyalgia could lead to a decrease in antioxidant capacity, with reduced activity of enzymes such as catalase, superoxide dismutase or enzymes involved in glutathione metabolism [24]. However, to the best of our knowledge, only one study has been focused on investigating the acute effects of exercise on oxidative stress markers. Santos, et al. [25] showed that a single trial of whole-body vibration exercise improved oxidant and antioxidant parameters, improving their adaptation to the stress response in women with FM.

Thus, there is a need for studies that investigate the acute effect of exercise on autonomic modulation and salivary markers. In this study, we aimed to investigate the differences between people with FM and healthy controls on HRV and salivary parameters (such as flow, protein concentration, enzymatic activities of amylase, catalase and glutathione peroxidase) in two moments: (1) at baseline, and (2) after an exercise fatigue protocol. We hypothesized that people with FM will exhibit more abnormal autonomic modulation and salivary biomarkers than healthy controls. In addition, immediately after exercise, a significantly different pattern in the HRV will be found as well as differences in the salivary biomarkers concentration between people with FM and healthy controls. Results would be relevant since it will constitute a differentiated strategy in order to identify salivary markers with potential use in the diagnosis of fibromyalgia.

## 2. Materials and Methods

### 2.1. Participants

Taking into account the sample size calculation performed with the G*Power soft-ware 3.1.9.4 (Kiel University, Kiel, Germany), a minimum of 16 people per group were needed to achieve a 90% of power to detect significant differences with an alpha of 0.001, using the Mann–Whitney U test. For sample size calculation, data was extracted from a previous study from Figueroa, et al. [26], where people with fibromyalgia exhibited an RMSSD of 2.9 (0.8), and healthy controls an RMSSD of 3.5 (0.5). In addition, 25% more of people with fibromyalgia were recruited in order to ensure the minimum sample size required. Therefore, a total of 37 participants were enrolled in the study. Twenty-one were people with fibromyalgia and sixteen were healthy controls. People with fibromyalgia were recruited in the family health unit “LUSITANIA” of Évora (Portugal). Healthy controls were recruited by an advertisement in the University of Évora. The last participant was enrolled on the date of April 2021.

People with fibromyalgia had a mean age (and a standard deviation) of 51.14 (9.99) years old and the healthy control group a mean age of 42.25 (13.15). People with fibromyalgia had a mean height of 158.95 (8.58) cm and a weight of 80.34 (22.82) kg, whereas the healthy control group had a mean height of 163.81 (4.50) cm and a weight of 61.32 (7.16) kg. The body mass index (BMI) for people with fibromyalgia is 31.61 (7.61) and 22.96 (3.49) for healthy controls. Eight participants from the fibromyalgia group were not included in the HRV analysis due to a problem with the HRV register. In the biochemist variables, some outliers were identified for each variable. These outliers were removed from analyses (three from people with fibromyalgia and two from the healthy control group).

All procedures were conducted following the Helsinki Declaration (revised in Brazil, 2013) and approved by the University research ethic committee (GD/44902/2019).

### 2.2. Procedure

A general warm-up consisted of three minutes of cycling (Monark Exercise AB, Vansbro, Sweden) at 50–60 rpm with no resistance to avoid fatigue. Then, a specific warm-up consisted of three repetitions of knee extensions and flexions of the dominant leg without resistance, and free velocity with a Biodex System 3 (Biodex Corporation, Shirley, NY, USA) was conducted. Lastly, the exercise fatigue protocol consisted of 20 repetitions of knee extensions and flexions of the dominant leg at 180 °·s^−1^ [27,28]. HRV and saliva samples were collected immediately before the general warm-up and immediately after the exercise fatigue protocol. All measurements were collected between 9:30 and 12:30 am. Participants were encouraged to avoid physical exercise 24 h before the evaluation.

### 2.3. Instruments, Processing and Outcomes

#### 2.3.1. Heart Rate Variability

A chest band with an Ant+ connection to Golden Cheetah software was used to assess the HRV before and after the exercise fatigue protocol. Data was exported to Kubios software (v. 3.3) [29], which allowed for preprocessing and extracting time-domain, frequency domain and non-linear variables. Artefacts were filtered using a middle filter, which allowed for identifying those RR intervals shorter/longer than 0.25 s, compared to the average of the previous beats. A cubic spline interpolation was used to correct and replace the artefacts.

Time-domain, frequency-domain and non-linear measures were extracted. In the time domain, variables such as maximum heart rate (maximum HR), mean heart rate (mean HR), RR intervals, RR50 count divided by the total number of all RR ranges (Pnn50), the standard deviation of all RR intervals (SDNN) and the square root of differences between adjacent RR intervals (RMSSD). In the frequency domain, the very low frequency (VLF, 0.0033–0.04 Hz), low frequency (LF, 0.04–0.15 Hz), high frequency (HF, 0.15–0.4 Hz) and ratio (LF/HF) were calculated. Regarding non-linear measures, the RR variability from heartbeat to short-term Poincaré graph (width) (SD1), RR variability from heartbeat to long-term Poincaré graph (length) (SD2) and the Sample Entropy (SampEn) were extracted. Moreover, the stress index, the parasympathetic nervous system index (PNS index) and the sympathetic nervous system index (SNS index) were calculated. The stress index is the square root of the Baevsky’s stress. The PNS index was calculated based on the mean RR (ms), the RMSSD (ms) and the SD1 (%). The SNS index was calculated based on the mean HR (bpm), the Baevsky’s stress index and the SD2 (%).

#### 2.3.2. Salivary Biomarkers

Unstimulated whole saliva was collected at rest and after exercise for each participant by direct draining into an ice-cold collection tube (pre-weighted) for 3 min. Subjects refrained from eating and drinking for at least 1 h before collection. After saliva collection, tubes with the samples were weighted (for saliva flux evaluation, mL/min), centrifuged at 1500× *g* for 10 min to remove food and cell debris, and the supernatant was stored at −20 °C until analysis. Preceding analysis and saliva samples were thawed on ice and centrifuged for 30 min at 4 °C, 13,000× *g*, for removal of mucinous material [30]. Supernatant total protein concentration was assayed using the Bradford method [31].

Dinitrosalicylic acid assay was used for measuring the starch-hydrolyzing activity of salivary α -amylase, as described at [32], minimized for 96-wells microplates. The reaction mixture consisted of 1% starch solution and saliva sample (diluted to 10 μg protein/mL protein in 20 mM phosphate buffer (pH 7.0). After incubation at 37 °C for 20 min, the reaction was stopped by the addition of the DNS reagent. Samples were heated to 90 °C for 30 min. Further, sodium and potassium tartrate (40%) were added to samples. Absorbance was measured at 530 nm. The absorbance values were then converted to glucose equivalent using a standard curve (3–18 mM). The results were expressed as μmol glucose/min/mg salivary protein.

Catalase activity was determined following the consumption of H_2_O_2_ at 240 nm, measuring absorbance every 30 s for 6 min. Reactional mixture consisted in salivary samples diluted in 10 mM potassium phosphate buffer (pH 7.0) and 0.2% H_2_O_2_. Enzyme activity was expressed in μmol of H_2_O_2_ consumed per minute per mg of salivary protein [33].

Glutathione peroxidase activity was determined following the oxidation of NADPH at 340 nm, using *t*-butylhydroperoxid as substrate. Absorbance measurements were performed every 30 s for 6 min. Enzyme activity was expressed in μmol of NADPH consumed per minute per mg of salivary protein [34].

#### 2.3.3. Borg Scale

The rating of perceived exertion (RPE) on a 6–20 scale was evaluated [35]. The RPE was asked immediately after the conclusion of the exercise fatigue protocol.

### 2.4. Statistical Analysis

The IBM SPSS (Statistical Package for Social Sciences, version 25) statistical software was employed to conduct the analyses. Post-exercise data was normalized taking into account the baseline values (subtracting the post exercise value to the baseline value). The Mann–Whitney U revealed significant differences between healthy controls and people with fibromyalgia in age and BMI. Therefore, these variables (age and BMI) were used as covariates when analyzing group differences. Thus, ANCOVA was employed to investigate between group differences. Differences within groups (comparison between pre and post values) were investigated using the Wilcoxon signed-rank test. The effect size, eta partial square and r were calculated for ANCOVA and Wilcoxon signed-rank test, respectively. Effects sizes were classified as follows: >0.5 is a large effect, between 0.5 and 0.3 a medium effect, and <0.3 is considered a small effect [36,37].

## 3. Results

### 3.1. Differences between People with Fibromyalgia and Healthy Controls at Baseline

Significant differences between people with fibromyalgia and healthy controls were found in age (*p*-value = 0.024) and BMI (*p*-value < 0.001). Therefore, these variables (age and BMI) have been used as covariates when analyzing between group differences.

Table 1 shows the differences between people with fibromyalgia and healthy controls in the HRV. Significant differences were found in the stress index (*p*-value = 0.014), LF (*p*-value = 0.047) and HF (*p*-value = 0.010).

**Figure 1:**
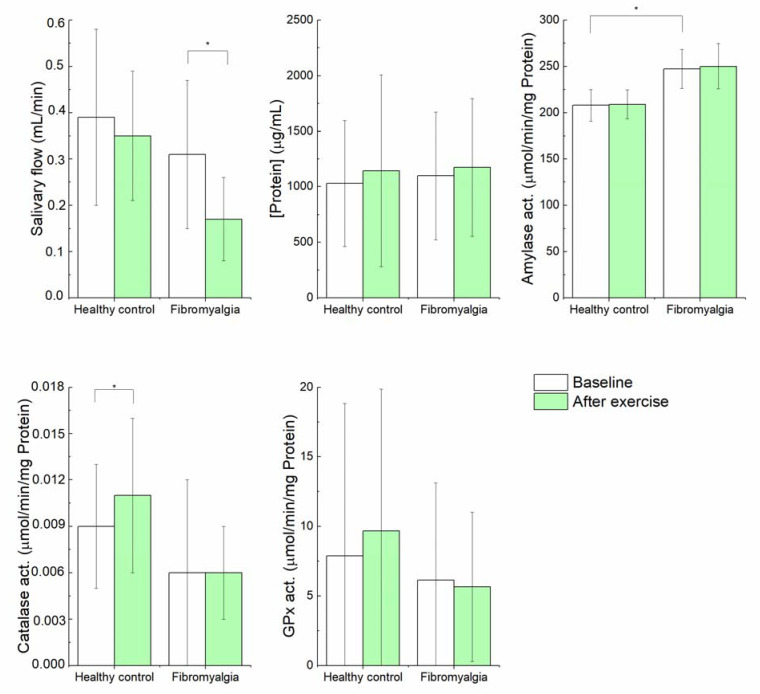
Differences in salivary biomarkers in fibromyalgia and heathy control groups, in baseline (white bars) and after physical exercise (green bars); * *p* < 0.05; (mean ± SD).

Regarding biochemist variables (Figure 1—white bars and Supplementary Table S1), significantly higher values of amylase (µmol/min/mg) (*p*-value 0.001) was found in people with FM relative to healthy controls.

### 3.2. Differences between Pre and Post Physical Exercise in Fibromyalgia and Healthy Controls Groups

Table 2 shows the differences in HRV between baseline and post-exercise in people with fibromyalgia. The Mann–Whitney U test revealed significant differences in the maximum HR (*p*-value = 0.002), mean HR (*p*-value = 0.009), RR interval (*p*-value = 0.006) and SampEn (*p*-value = 0.006).

Differences in salivary biomarkers between baseline and post-exercise in people with fibromyalgia are depicted in Figure 1 and in Supplementary Table S2. The Mann–Whitney U test revealed a significant decrease of salivary flow (*p*-value = 0.001) after physical exercise.

Table 3 shows the differences in HRV between baseline and post-exercise in healthy controls. The Mann-Whitney U test showed significant differences in the maximum HR (*p*-value = 0.001), mean HR (*p*-value = 0.003), RR interval (*p*-value = 0.002), PNS index (*p*-value = 0.004), SNS index (*p*-value = 0.010), LF (*p*-value = 0.020), HF (*p*-value = 0.026) and LF/HF (*p*-value = 0.038).

Differences in salivary biochemical variables between baseline and post-exercise in people with fibromyalgia are shown in Figure 1 and in the Supplementary Table S3. The Mann–Whitney U test revealed a significant increase of catalase activity (µmol/min/mg) (*p*-value = 0.047) after physical exercise.

### 3.3. Acute Effects of Exercise on HRV and Salivary Biomarkers

Table 4 shows the impact of physical exercise on normalized HRV of people with FM and healthy controls. Differences between groups were not found in any of the HRV or salivary biomarkers included in the present study.

### 3.4. RPE after Exercise Protocol

RPE differences between people with FM and healthy controls are shown in the Supplementary Table S4. Significant differences were found in the RPE at baseline and after exercise, with higher values corresponding to people with fibromyalgia. In addition, both groups significantly increased the RPE after exercise.

## 4. Discussion

In this study, we aimed to investigate the differences between people with FM and healthy controls on HRV and salivary parameters (such as flow, protein concentration, enzymatic activities of amylase, catalase and glutathione peroxidase) in two moments: (1) at baseline, and (2) after an exercise fatigue protocol. At baseline, significant differences were found in the HRV (stress index, LF and HF variables) and salivary biomarkers (with higher concentration of salivary amylase in people with FM compared to healthy controls). Exercise acute effects on HRV showed that people with FM did not significantly react to exercise. However, significant differences between baseline and post-exercise on HRV significantly induce alteration on the HRV of healthy controls. Catalase significantly increased after exercise in healthy controls whereas salivary flow significantly increased in women with FM after an exercise fatigue protocol.

Our results showed significant differences between people with FM and the control group. In this regard, people with FM exhibited higher values of Stress Index and LF as well as lower values of HF than healthy controls. It is believed that increments in these indices are related to the sympathetic modulation [38]. Furthermore, salivary α-amylase activity is higher in people with FM than in the control group. This enzyme is one of the most abundant proteins in saliva, playing an important role in starch digestion [39]. It is secreted by the acinar cells of the salivary glands in response to stimuli from the autonomic nervous system, especially from the sympathetic branch, and the increase in the activity of this enzyme in saliva is considered a biomarker of adrenergic activation [39,40,41,42]. Several articles reported higher α-amylase activity in people with fibromyalgia relatively to healthy subjects [18,43,44,45,46], revealing a sympathetic hyperreactivity, so our results corroborate those previously described.

Catalase is an antioxidant enzyme that plays the important role of degrading H_2_O_2_ to H_2_O, contributing to the maintenance of an adequate oxidative environment in living systems. This enzyme is not secreted by the salivary glands so its concentration in saliva reflects its concentration in blood [19]. Previous studies showed a lower catalase activity in blood in people with fibromyalgia than in healthy people [47,48,49]. This could indicate that people with FM have more susceptibility to the deleterious effects of ROS. However, in our study, we did not find significant differences.

The complex interplay between the autonomic nervous system and the cardiovascular system during exercise can provide significant prognostic information [14]. Thus, people with FM and healthy controls were exposed to an exercise fatigue protocol. Regarding HRV, the control group significantly increased the maximum and average HR, SNS index, LF and HF/LF and reduced the RR, PNS index and HF. People with FM showed a significant change in the maximum HR, average HR, RR interval and LF. However, the variation was attenuated compared to the control group, especially the maximum HR, which varies 25% in control and 6% in patients with fibromyalgia. However, perceived exertion results revealed that both groups significantly increase their RPE. These results can be explained by the dysautonomia that people with FM exhibited. Dysautonomia consisted of persistent autonomic nervous system hyperactivity at rest and hyperreactivity during stressful situations [12]. In the same line, previous studies observed dysautonomia of people with FM could lead to chronotropic incompetence, and the inability to increase heart rate with increasing exercise intensities [50,51].

Regarding the parameters measured in saliva, a significant increase, induced by exercise, was found in the catalase activity of control group. Salivary parameters such as flow, total protein concentration and α-amylase activity, dependent on ANS stimuli in the salivary glands [52], did not show significant changes after the fatigue exercise protocol. Actually, changes in salivary biochemical parameters seems to be dependent on the type and intensity of the physical exercise [53,54,55]. For instance, total protein and α-amylase activity tends to increase immediately after very intense exercise [53,56,57,58,59], but not with moderate exercise [58]. RPE values obtained in both control and people with FM ranged from 12.75 (3.34) to 14.69 (3.17), respectively, which corresponds to light to somewhat hard effort [35]. In this line, changes observed in the control group with the exercise in HRV parameters, either in the time component or in the frequency component, show us that the exercise performed required effort, although the associated adrenergic activation was not enough to induce significant changes in salivary secretion. The reported increase in catalase activity may reflect the increase in this activity in the blood with exercise [49].

Comparing salivary parameters variations after the exercise fatigue protocol in people with FM and controls, the more significant alteration was found in the salivary flow, which reduces 45.16% in people with fibromyalgia and did not show significant variation in control. Thus, it can be deduced that a stronger parasympathetic nervous system inhibition occurred more in people with fibromyalgia than in healthy individuals. As our HRV also supported, there is evidence that the sympathetic nervous system is already more active than the parasympathetic nervous system in people with fibromyalgia compared to controls even at baseline [13,60]. However, in people with FM, catalase activity did not change after the fatigue exercise protocol, as observed in the control group. Perhaps the reduction in the salivary flow, which occurs by inhibition of the PNS [22,53,61], did not promote the transfer of enzymes from the blood to the saliva, justifying similar salivary catalase activity before and after the fatigue exercise protocol in people with fibromyalgia. Indeed, Soliman, El-Olemy, Hassan, Shaker and Abdullah [49] showed a higher catalase activity in the blood after exercise in people with fibromyalgia.

About glutathione peroxidase (GPx), an antioxidant enzyme that is part of the glutathione metabolism responsible for neutralizing H_2_O_2_ with the concomitant oxidizing of glutathione-to-glutathione disulfide, no significative differences between or within groups were found. Reduced concentration in thiol-rich compounds (including glutathione) was described in fibromyalgia blood samples [47,49]. Additionally, in red blood cells from people with fibromyalgia, a lower GPx activity was found [48]. These results also underline the lower antioxidative capacity of people with fibromyalgia [17,47,48,49,61,62]. Nevertheless, GPx activity in saliva is not a good marker of this feature, probably due to a very low salivary activity of this enzyme.

This study has some limitations that should be acknowledged. Firstly, differences between groups at baseline were detected for age and BMI. Although these variables have been included in the analysis through ANCOVA, a potential bias could still remain. Nevertheless, our study followed the line of a previous study which analyzed the physiological response to exercise of people with fibromyalgia and healthy controls [63]. However, results might be taken with caution and future studies should confirm our results. Secondly, the exercise proposed in the present study was focused on muscle fatigue. Future studies should evaluate if these results are repeated when using a general fatigue protocol such as a walking test. We did not consider the assessment of myalgic encephalomyelitis/chronic fatigue syndrome, which we believe should be taken into account in future studies. As a future for the assessment of the ANS, a broader analysis of small sensory fibers, including sudometry and laser evoked potentials, as well as the diagnosis of Sicca Syndrome, should be associated. When evaluating salivary flow and the biochemical markers associated with saliva, we can consider a limitation that we did not perform biopsies of accessory salivary glands.

## 5. Conclusions

Our study suggests that a higher α-amylase activity and an impaired HRV can be used as possible biomarkers of fibromyalgia, associated with a reduction in salivary flow without changes in HRV and catalase activity after a fatigue exercise protocol. More studies should be carried out in the future to evaluate this hypothesis, in order to find diagnostic biomarkers in fibromyalgia.

## Figures and Tables

**Table 1:** Differences in Heart Rate Variability between people with fibromyalgia and healthy controls at baseline.

Variable	Fibromyalgia Mean (SD)	Healthy Controls Mean (SD)	*p*-Value	F	Effect Size
Maximum HR	82.29 (12.08)	87.69 (9.01)	0.615	0.257	0.008
mean HR	74.86 (10.68)	76.81 (7.88)	0.950	0.004	<0.001
RR	818.43 (125.90)	788.94 (81.10)	0.831	0.046	0.001
SDNN	30.35 (20.63)	51.67 (30.61)	0.158	2.088	0.060
pNN50	9.25 (14.07)	22.18 (17.68)	0.248	1.383	0.040
RMSSD	26.93 (17.64)	55.12 (35.09)	0.059	3.832	0.104
Stress Index	17.11 (7.13)	10.85 (4.07)	0.014	6.782	0.170
PNS Index	−2.93 (9.22)	−0.06 (1.22)	0.473	0.528	0.016
SNS Index	1.77 (1.60)	0.86 (0.95)	0.065	3.642	0.099
VLF	11.59 (9.59)	4.68 (3.15)	0.234	1.473	0.043
LF	60.56 (18.44)	45.98 (18.98)	0.047	4.274	0.115
HF	27.21 (15.93)	49.12 (20.38)	0.010	7.375	0.183
LF/HF	4.82 (9.17)	1.49 (1.54)	0.181	1.869	0.054
SD1	19.08 (12.52)	39.06 (24.86)	0.059	3.837	0.104
SD2	38.08 (27.03)	60.65 (37.46)	0.250	1.373	0.040
SampEn	1.67 (0.36)	1.61 (0.39)	0.737	0.115	0.003

Maximum HR: The heart rate peak obtained in the register; Mean HR: the average heart rate obtained in the register; RR: Time between intervals R-R; SDNN: standard deviation of all RR intervals; pNN50: Percentage of intervals >50 ms different from the previous interval; RMSSD: the square root of the mean of the squares of the successive differences of the interval RR; VLF: Very low frequency; LF/HF: Low frequency (LF) ratio (ms2)/High frequency (HF) (ms2); Total power: The sum of all the spectra; PNS index: Parasympathetic nervous system index, SNS index: Sympathetic nervous system index and stress index; SD1: Dispersion, standard deviation of points perpendicular to the axis of line-of-identity in the Poincaré plot; SD2: Dispersion, standard deviation of points along the axis of line-of-identity in the Poincaré plot; SampEn: Sample Entropy.

**Table 2:** HRV at baseline and after exercise in people with fibromyalgia.

Variable	Baseline Mean (SD)	Post-Exercise Mean (SD)	*p*-Value	Z	Effect Size
Maximum HR	82.29 (12.08)	87.62 (12.18)	0.002	−3.114	0.864
mean HR	74.86 (10.68)	75.77 (9.47)	0.009	−2.609	0.724
RR	818.43 (125.90)	803 (108.45)	0.006	−2.726	0.756
SDNN	30.35 (20.63)	31.15 (10.74)	0.196	−1.293	0.359
pNN50	9.25 (14.07)	5.41 (5.94)	0.944	−0.070	0.019
RMSSD	26.93 (17.64)	29.31 (16.03)	0.421	−0.804	0.223
Stress Index	17.11 (7.13)	13.68 (4.87)	0.108	−1.609	0.446
PNS Index	−2.93 (9.22)	−0.87 (0.73)	0.944	−0.070	0.019
SNS Index	1.77 (1.60)	1.30 (1.08)	0.753	−0.315	0.087
VLF	11.59 (9.59)	9.82 (5.46)	0.055	−1.922	0.533
LF	60.56 (18.44)	62.68 (15.32)	0.028	−2.201	0.610
HF	27.21 (15.93)	27.34 (15.70)	0.249	−1.153	0.320
LF/HF	4.82 (9.17)	4.64 (5.33)	0.075	−1.782	0.494
SD1	19.08 (12.52)	20.75 (11.33)	0.421	−0.804	0.223
SD2	38.08 (27.03)	38.02 (13.13)	0.184	−1.328	0.368
SampEn	1.67 (0.36)	1.47 (0.42)	0.006	−2.760	0.765

Maximum HR: The heart rate peak obtained in the register; Mean HR: the average heart rate obtained in the register; RR: Time between intervals R-R; SDNN: standard deviation of all RR intervals; pNN50: Percentage of intervals >50 ms different from the previous interval; RMSSD: the square root of the mean of the squares of the successive differences of the interval RR; VLF: Very low frequency; LF/HF: Low frequency (LF) ratio (ms2)/High frequency (HF) (ms2); Total power: The sum of all the spectra; PNS index: Parasympathetic nervous system index, SNS index: Sympathetic nervous system index and stress index; SD1: Dispersion, standard deviation of points perpendicular to the axis of line-of-identity in the Poincaré plot; SD2: Dispersion, standard deviation of points along the axis of line-of-identity in the Poincaré plot; SampEn: Sample Entropy.

**Table 3:** HRV at baseline and after exercise in healthy controls.

Variable	Baseline Mean (SD)	Post-Exercise Mean (SD)	*p*-Value	Z	Effect Size
Maximun HR	87.69 (9.01)	116.69 (20.25)	0.001	−3.408	0.852
mean HR	76.81 (7.88)	87.31 (11.38)	0.003	−3.004	0.751
RR	788.94 (81.10)	696.62 (85.06)	0.002	−3.051	0.763
SDNN	51.67 (30.61)	45.56 (25.42)	0.352	−0.931	0.233
pNN50	22.18 (17.68)	15.11 (16.41)	0.352	−0.931	0.233
RMSSD	55.12 (35.09)	40.16 (24.54)	0.109	−1.603	0.401
Stress Index	10.85 (4.07)	11.42 (3.82)	0.485	−0.698	0.175
PNS Index	−0.06 (1.22)	−1.09 (0.99)	0.004	−2.844	0.711
SNS Index	0.86 (0.95)	1.74 (1.25)	0.010	−2.585	0.646
VLF	4.68 (3.15)	5.52 (5.42)	0.485	−0.698	0.175
LF	45.98 (18.98)	63.27 (15.81)	0.020	−2.327	0.582
HF	49.12 (20.38)	31.07 (16.15)	0.026	−2.223	0.556
LF/HF	1.49 (1.54)	3.07 (2.34)	0.038	−2.068	0.517
SD1	39.06 (24.86)	28.42 (17.38)	0.109	−1.603	0.401
SD2	60.65 (37.46)	57.61 (31.91)	0.717	−0.362	0.091
SampEn	1.61 (0.39)	1.55 (0.29)	0.408	−0.827	0.207

Maximum HR: The heart rate peak obtained in the register; Mean HR: the average heart rate obtained in the register; RR: Time between intervals R-R; SDNN: standard deviation of all RR intervals; pNN50: Percentage of intervals >50 ms different from the previous interval; RMSSD: the square root of the mean of the squares of the successive differences of the interval RR; VLF: Very low frequency; LF/HF: Low frequency (LF) ratio (ms2)/High frequency (HF) (ms2); Total power: The sum of all the spectra; PNS index: Parasympathetic nervous system index, SNS index: Sympathetic nervous system index and stress index; SD1: Dispersion, standard deviation of points perpendicular to the axis of line-of-identity in the Poincaré plot; SD2: Dispersion, standard deviation of points along the axis of line-of-identity in the Poincaré plot; SampEn: Sample Entropy.

**Table 4:** Impact of exercise on HRV and on salivary biomarkers in people with fibromyalgia and healthy controls.

Variable	Fibromyalgia	Healthy Controls	*p*-Value	F	Effect Size
Heart Rate Variability					
Maximun HR	8.15 (5.47)	29 (19.81)	0.130	2.452	0.089
mean HR	3.69 (4.59)	10.50 (10.20)	0.501	0.467	0.018
RR	−41.15 (46.42)	−92.31 (89.16)	0.564	0.342	0.013
SDNN	5.61 (12.34)	−6.12 (22.67)	0.261	1.320	0.050
pNN50	0.12 (5.16)	−7.07 (18.21)	0.381	0.795	0.031
RMSSD	5.11 (16.90)	−14.96 (32.86)	0.277	1.233	0.047
Stress Index	−3.13 (6.71)	0.57 (3.48)	0.184	1.863	0.069
PNS Index	3.21 (11.79)	−1.03 (1.23)	0.355	0.887	0.034
SNS Index	−0.22 (1.30)	0.88 (1.08)	0.172	1.981	0.073
VLF	−5.44 (8.07)	0.84 (4.04)	0.148	2.227	0.082
LF	11.63 (17.42)	17.28 (23.49)	0.996	<0.001	<0.001
HF	−5.35 (17.86)	−18.05 (24.67)	0.575	0.323	0.013
LF/HF	2.59 (4.77)	1.58 (2.61)	0.188	1.836	0.068
SD1	3.61 (11.95)	−10.64 (23.27)	0.275	1.244	0.047
SD2	6.55 (14.03)	−3.04 (26.11)	0.349	0.911	0.035
SampEn	−0.27 (0.28)	−0.05 (0.33)	0.241	1.440	0.054
**Salivary Biomarkers**					
Salivary flow (mL/min)	−0.14 (0.14)	−0.04 (0.12)	0.114	2.644	0.081
Proteins (µg/mL)	75.45 (343.87)	114.99 (387.30)	0.551	0.363	0.012
Amylase (µmol/min/mg)	2.62 (19.50)	1.07 (24.39)	0.427	0.652	0.025
Catalase (µmol/min/mg)	0.0003 (0.004)	0.002 (0.004)	0.941	0.006	<0.001
GPx (µmol/min/mg)	−0.21 (5.55)	1.80 (6.72)	0.518	0.429	0.015

Maximum HR: The heart rate peak obtained in the register; Mean HR: the average heart rate obtained in the register; RR: Time between intervals R-R; SDNN: standard deviation of all RR intervals; pNN50: Percentage of intervals >50 ms different from the previous interval; RMSSD: the square root of the mean of the squares of the successive differences of the interval RR; VLF: Very low frequency; LF/HF: Low frequency (LF) ratio (ms2)/High frequency (HF) (ms2); Total power: The sum of all the spectra; PNS index: Parasympathetic nervous system index, SNS index: Sympathetic nervous system index and stress index; SD1: Dispersion, standard deviation of points perpendicular to the axis of line-of-identity in the Poincaré plot; SD2: Dispersion, standard deviation of points along the axis of line-of-identity in the Poincaré plot; SampEn: Sample Entropy; GPx: Gluthatione Peroxidase.

## Data Availability

Not applicable.

## Supplementary Materials

The following supporting information can be downloaded at: https://www.mdpi.com/article/10.3390/diagnostics12092220/s1, Table S1. Salivary biomarkers in FM and healthy control groups at baseline; Table S2. Biochemist variables at baseline and after exercise in people with fibromyalgia; Table S3. Biochemist variables at baseline and after exercise in healthy controls; Table S4. Differences between fibromyalgia and healthy controls in Borg scale at baseline and post-exercise.
