# Supplementary material for: Heart Rate Variability and Salivary Biomarkers Differences between Fibromyalgia and Healthy Participants after an Exercise Fatigue Protocol: An Experimental Study
Source: Diagnostics (Basel). 2022 Sep 14;12(9):2220. doi: 10.3390/diagnostics12092220 (PMC9497903; doi:10.3390/diagnostics12092220)
Supplement: Supplementary file 1 [file diagnostics-12-02220-s001.zip › diagnostics-1860253-supplementary.pdf]

Supplementary material

Supplementary Table S1 shows the differences in salivary biomarkers between FM and Healthy control groups. Mann-Whitney test revealed that FM group presented higher α -amylase activity (μmol/min/mg) (p = 0.001) over control group.

Supplementary Table S1. Salivary biomarkers in FM and Healthy control groups at baseline

Variable	Fibromyalgia Mean (SD)	Healthy controls Mean (SD)	p-value	Z	Effect Size
Salivary flow (mL/min)	0.31 (0.16)	0.39 (0.19)	0.314	0.928	0.085
Proteins (μg/mL)	1098.39 (573.87)	1028.72 (566.55)	0.113	2.664	0.079
α -amylase (μmol/min/mg)	247.41 (20.96)	207.98 (17.10)	0.001	15.388	0.372
Catalase (μmol/min/mg)	0.006 (0.006)	0.009 (0.004)	0.190	1.804	0.059
Glutathione Peroxidase (μmol/min/mg)	6.13 (6.99)	7.87 (10.97)	0.415	0.684	0.022

Supplementary Table S2 shows the differences in biochemical variables between baseline and post-exercise in people with fibromyalgia. Mann-Whitney test revealed a significant decrease of salivary flow (p-value=0.001) after physical exercise.

Supplementary Table S2. Salivary biomarkers variables at baseline and after exercise in people with fibromyalgia

Variable	Baseline Mean (SD)	Post-Exercise Mean (SD)	p-value	Z	Effect Size
Salivary flow (mL/min)	0.31 (0.16)	0.17 (0.09)	0.001	-3.250	0.709
Proteins (μg/mL)	1098.39 (573.87)	1173.84 (618.99)	0.332	-0.971	0.217
Amylase (μmol/min/mg)	247.41 (20.96)	250.18 (24.47)	0.587	-0.543	0.125
Catalase (μmol/min/mg)	0.006 (0.006)	0.006 (0.003)	0.492	-0.686	0.166
Glutathione Peroxidase (μmol/min/mg)	6.13 (6.99)	5.65 (5.36)	0.868	-0.166	0.040

Supplementary Table S3 shows the differences in biochemical variables between baseline and post-exercise in people with fibromyalgia. Mann-Whitney test revealed a significant increase of catalase activity (µmol/min/mg) (p-value=0.047) after physical exercise.

Supplementary Table S3. Salivary biomarkers at baseline and after exercise in healthy controls

**Variable**	**Baseline Mean (SD)**	**Post-Exercise Mean (SD)**	**p-value**	**Z**	**Effect Size**
Salivary flow (mL/min)	0.39 (0.19)	0.35 (0.14)	0.249	-1.153	0.320
Proteins (µg/mL)	1028.72 (566.55)	1143.72 (864.01)	0.427	-0.795	0.205
Amylase (µmol/min/mg)	207.98 (17.10)	209.04 (15.66)	0.878	-0.153	0.048
Catalase (µmol/min/mg)	0.009 (0.004)	0.011 (0.005)	0.047	-1.988	0.513
Glutathione Peroxidase (µmol/min/mg)	7.87 (10.97)	9.67 (10.21)	0.256	-1.136	0.293

Supplementary Table S4 shows the RPE differences between people with FM and healthy controls. Significant differences were found in the RPE at baseline and after exercise, with higher values corresponding to people with fibromyalgia. In addition, both groups significantly increased the RPE after exercise.

Supplementary Table S4. Differences between fibromyalgia and healthy controls in Borg scale at baseline and post-exercise.

	Baseline Borg (6-20)	Post Borg (6-20)	Within group comparison
	Mean (SD)	Mean (SD)	p-value
Fibromyalgia group	10.38 (2.99)	14.69 (3.17)	0.003
Healthy control group	6 (00)	12.75 (3.34)	<0.001
Between groups comparison (p-value)	<0.001	0.371	
